# Complete Genome Sequence of Escherichia coli Bacteriophage U136B

**DOI:** 10.1128/MRA.00030-21

**Published:** 2021-04-01

**Authors:** A. R. Burmeister, Denish Piya, Paul E. Turner

**Affiliations:** a Department of Ecology and Evolutionary Biology, Yale University, New Haven, Connecticut, USA; b BEACON Center for the Study of Evolution in Action, East Lansing, Michigan, USA; c Department of Bioengineering, University of California, Berkeley, Berkeley, California, USA; d Program in Microbiology, Yale School of Medicine, New Haven, Connecticut, USA; Queens College CUNY

## Abstract

We report the genome sequence of bacteriophage U136B, which is reliant on the lipopolysaccharide and the antibiotic efflux protein TolC for infection of Escherichia coli and is a useful model for studying trade-offs and trade-ups that shape evolution. Phage U136B has a 49,233-bp genome with 87 predicted genes.

## ANNOUNCEMENT

Phage U136B is a useful model for studying evolutionary trade-offs and trade-ups that shape the evolution of bacterial populations (1, 2).

Bacteriophages were cultured on Escherichia coli growing on LB at 37°C with aeration. Bacteriophage U136B DNA was extracted using a phage DNA isolation kit (Norgen Biotek). The sequencing library was prepared using transposome-based tagmentation chemistry with the Nextera XT DNA kit (Illumina) and sequenced at the Yale Center for Genome Analysis on a MiSeq system to ∼1,800× coverage. Sequences were randomly rarified to a target of 100× coverage to improve phage genome assembly (3). Illumina adaptor sequences were removed with Cutadapt version 2.6 (4). Sequences were trimmed for quality using Sickle version 1.33 (5) and assembled with SPAdes version 3.13 (6). The resulting assembly had 108× coverage of 49,350 bp. One contig (comprising 470 bp at 0.95× coverage) was omitted from further analysis. The phage U136B genome has a circularly permuted genome, as determined by terminal repeats of 127 bp. After removal of these 127 bp, a final contig contained 49,223 bp. The assembly was validated using BWA version 7.17 (7), with 99.7% of the sequences mapping back to the assembly, above the 90% threshold typically considered valid (3). The complete genome sequence was reverse complemented and reopened to be syntenic with bacteriophage TLS (NCBI reference sequence NC_009540). All tools were run with default parameters unless otherwise specified.

Genome annotation was conducted using the Galaxy (8) and Web Apollo (9) platforms for phage genome annotation (10). Structural gene prediction was completed using GLIMMER version 3.0 (11), MetaGeneAnnotator version 1.0 (12), and SixPack (13). Structural predictions were manually and individually confirmed based on the assessment of ribosomal binding sites, translation start/stop sites, and gene overlaps. Gene functions were predicted using BLASTp (14, 15), and putative functions were then manually and individually assigned upon review of the BLASTp results. One gene putatively encoding a tape measure chaperone via a slipper sequence (16) was manually identified and annotated using the ExPASy Translate tool (17) and BLASTp at the NCBI (18). No tRNA genes were found using either tRNAscan-SE version 2.0 (19) or ARAGORN (20). Of 87 predicted open reading frames (ORFs), 43 putative functional proteins and 44 hypothetical proteins were annotated. No ORFs had predicted integrase functions, consistent with prior results showing that phage U136B is strictly lytic (2). The GC content was determined to be 43% using GeeCee (21, 22).

A whole-genome BLASTn search (18) indicated that phage U136B is in the *Tlsvirus* phage group, which includes E. coli bacteriophage TLS (GenBank accession number AY308796.1) (93% identity) and E. coli bacteriophage LL5 (NCBI reference sequence NC_047985.1) (94% identity), both of which are also reliant on the E. coli TolC efflux protein (23, 24) and have siphophage morphology with flexible tails (23, 25). Host range analysis of phage U136B indicates a limited host range of some, but not all, E. coli hosts (2).

### Data availability.

The annotated phage U136B genome has been deposited in NCBI GenBank under accession number MW598258. Original sequence reads have been deposited in the Sequence Read Archive (SRA) under SRA accession number SRR13337692 and BioProject accession number PRJNA688914.
